# From the logic of coordination to goal-directed reasoning: the agentic turn in artificial intelligence

**DOI:** 10.3389/frai.2025.1728738

**Published:** 2026-01-12

**Authors:** Tsehaye Haidemariam

**Affiliations:** BI Norwegian Business School, Oslo, Norway

**Keywords:** Agentic AI, artificial agency, autonomous systems, distributed intelligence, goal-directed reasoning

## Abstract

The rise of agentic artificial intelligence (Agentic AI) marks a transition from systems that optimize externally specified objectives to systems capable of representing, evaluating, and revising their own goals. Whereas earlier AI architectures executed fixed task specifications, agentic systems maintain recursive loops of perception, evaluation, goal-updating, and action, allowing them to sustain and adapt purposive activity across temporal and organizational scales. This paper argues that Agentic AI is not an incremental extension of large language models (LLMs) or autonomous agents in the sense we know it from classical AI and multi-agent systems, but a reconstitution of agency itself within computational substrates. Building on the logic of coordination, delegation, and self-regulation developed in early agent-based process management systems, we propose a general theory of synthetic purposiveness, where agency emerges as a distributed and self-maintaining property of artificial systems operating in open-ended environments. We develop the concept of synthetic teleology—the engineered capacity of artificial systems to generate and regulate goals through ongoing self-evaluation—and we formalize its dynamics through a recursive goal-maintenance equation. We further outline design patterns, computational semantics, and measurable indicators of purposiveness (e.g., teleological coherence, adaptive recovery, and reflective efficiency), providing a foundation for the systematic design and empirical investigation of agentic behaviour. By reclaiming agency as a first-class construct in artificial intelligence, we argue for a paradigm shift from algorithmic optimization toward goal-directed reasoning and purposive orchestration—one with far-reaching epistemic, societal, and institutional consequences.

## 1 Introduction

Artificial intelligence has long oscillated between two aspirations: the pursuit of cognition and the orchestration of control. Early AI research sought to emulate rational thought through symbolic reasoning, planning, and goal formation, while the later rise of machine learning emphasized pattern extraction and optimization. Despite their technical differences, both trajectories shared a common limitation: intelligence was largely treated as closed-loop computation rather than as an open-ended process of acting in the world. Recent developments in autonomous, self-directed systems—collectively termed *Agentic AI*—reopen this paradigm by foregrounding purposeful, context-sensitive action (Acharya et al., 2025; Botti, 2025). Although the term “Agentic AI” is newly fashionable, many of its core principles align with longstanding research on autonomous agents, defined as systems capable of autonomous, reactive, proactive, and socially coordinated behaviour (Wooldridge and Jennings, 1995).

The conceptual roots of Agentic AI are therefore best understood not through linear historical progression, but through a continuity of abstraction (Vu et al., 2026). Early agent-based process management systems, developed to coordinate distributed workflows and adaptive control, introduced the notion of autonomous yet cooperative entities (Jennings et al., 1996; Jennings et al., 1998a, 1998b; O’Brien and Wiegand, 1998). These systems instantiated a form of delegated intentionality: agents were designed not merely to execute instructions, but to interpret context, negotiate constraints, and dynamically modify behaviour (Ehrler et al., 2006; Kuo, 2004; Tony et al., 2005; Zhang et al., 2010). What began as a strategy for managing organizational complexity has, in contemporary AI architectures, evolved into a meta-structural principle—the embedding of purposive behaviour within computational substrates (Dawid and LeCun, 2023; Vu et al., 2026). This shift can be summarized as a movement:

- from reactive outputs to reflective, goal-directed reasoning
- from externally imposed objectives to self-maintained purpose
- from isolated computational modules to negotiating multi-agent ecologies.

Table 1 provides a conceptual contrast between classical AI and Agentic AI across key dimensions of autonomy, reasoning, coordination, and teleology/purposiveness.

**Table 1:** Comparative overview of classical AI and Agentic AI.

Dimension	Classical AI	Agentic AI	What changes/why it matters
Mode of intelligence	Reactive, task-bound output generation	Reflective, deliberative, goal-seeking cognition	From responding to *reasoning*
Purpose/teleology	Executes externally defined instructions	Forms, maintains, and revises goals	AI becomes *purposive*
Autonomy	Procedural automation	Self-directed, adaptive autonomy	Independence shifts from behaviour → intention
Context handling	Static input–output mappings	Dynamic sense-making and context modelling	Moves beyond pattern matching
Learning and reflection	Model-centric learning	Self-evaluation, meta-learning, recursive improvement	“Optimizing optimization”
Coordination/sociality	Isolated or sequential modules	Multi-agent negotiation and shared intentionality	Enables *collective intelligence*
Time horizon	Short horizon/episodic	Long-horizon/temporally extended planning	Action linked to continuity and memory
Governance	Control-and-compliance paradigm	Co-evolutionary alignment and value negotiation	Shifts the role of oversight

The distinction between “responding” (classical AI) and “reasoning” (Agentic AI) in Table 1 is not intended to deny the long tradition of symbolic reasoning, knowledge representation, and automated inference in classical artificial intelligence (McCarthy, 1980; Newell and Simon, 1976; Norvig and Russell, 2021). Systems based on logic, rule-based inference, planning, and theorem proving have supported sophisticated forms of formal reasoning for decades (Nilsson, 1980, 1998). The distinction instead concerns the *locus and reflexivity* of reasoning. In classical AI systems, reasoning typically operates as a task-bounded, externally triggered process over fixed representations, serving goals specified outside the system (Laird, 2012; Wooldridge, 2009). In Agentic AI systems, by contrast, reasoning becomes internally triggered and reflexive, applied not only to the environment but also to the system’s own goals, plans, and evaluative criteria, and embedded within continuous loops of goal maintenance and revision (Shinn et al., 2023).

In this sense, the shift from “responding” to “reasoning” refers not to the presence or absence of inference, but to a transition from instrumental reasoning about actions to meta-reasoning about purposes, priorities, and commitments (Nisa et al., 2025). Table 1 should therefore be read as contrasting externally framed task reasoning with internally regulated purposive reasoning, rather than as dismissing classical AI’s contributions to logical inference and planning.

This transformation has been accelerated by the rise of large foundation models and cognitive orchestration frameworks capable of sustained reasoning, planning, and adaptive tool use (Shinn et al., 2023; Yao et al., 2023). Whereas traditional AI systems required explicit task specifications, agentic systems increasingly construct and revise objectives in real time, guided by internal representations of both goal states and affordances (Pedrola and Vitari, 2025; Kuss and Meske, 2025). In doing so, they approach a form of *synthetic autonomy*: a dynamic capacity to align means and ends without continuous external supervision. Such autonomy does not imply consciousness or sentience; rather, it reflects operational closure, whereby systems maintain coherence across shifting contexts (Beer, 1995; Maturana and Varela, 1980; Botti, 2025).

The emergence of Agentic AI thus re-centres the foundational question of purpose in artificial systems. If machine learning represents the science of correlation, and deep learning the engineering of abstraction, then Agentic AI may be understood as a theory of artificial purposiveness (Dattathrani and De, 2023; Sapkota et al., 2026). Its architectures integrate learning, reasoning, and action into temporally extended feedback loops, enabling systems to pursue outcomes refined through iterative self-evaluation rather than fixed external metrics (Sapkota et al., 2026). Such systems embody what may be called *goal realism*: the recognition that intelligence unfolds not through static optimization, but through continuous negotiation between intention and environment (Gahnberg, 2021; Gershman et al., 2015).

Importantly, this shift also reconfigures the human–machine relation. Traditional automation displaced human labour by codifying routines; agentic systems, by contrast, operate within shared cognitive ecologies, collaborating as co-intentional partners that reason, negotiate, and self-correct within collective systems of meaning (Gershman et al., 2015). In this sense, Agentic AI is as much a socio-technical transformation as a computational one, inviting renewed consideration of governance, responsibility, and epistemic agency (Gahnberg, 2021; Gangavarapu, 2025; Leonardi, 2025; Shavit et al., 2023).

This paper advances the thesis that Agentic AI is not a new field of artificial intelligence, but AI recalling its original vocation: to build systems that act as well as reason. We argue that the core of this transformation lies not in algorithmic sophistication alone, but in the recovery of agency as a first-class computational construct. By tracing the structural logic of agentic architectures—from early process management paradigms to contemporary generative–cognitive systems—we develop a unified framework in which coordination, cognition, and autonomy converge. The remainder of the paper elaborates this claim across three domains: (1) the conceptual foundations of agentic architectures; (2) the emergence and formalization of synthetic purposiveness; and (3) the societal and institutional implications of distributed agency in human–AI systems.

## 2 Related work: teleology, function, and artificial artifacts

The concept of *teleology*—the explanation of systems in terms of purposes, ends, or goal-directedness—has a long and contested history in philosophy and the sciences. Classical treatments distinguish between goal-directed behaviour, which can be explained mechanistically, and teleological explanation, which appeals to the functional organization of a system (Nagel, 1961). Within contemporary philosophy of biology and technology, this has given rise to multiple accounts of *function*, including causal-role theories, etiological (proper function) theories, and intentionalist accounts (Chaigneau and Puebla, 2013; Griffiths, 1993).

Causal-role theories define a function in terms of the contribution a component makes to the capacities of a system (Cummins, 1975). On this view, a function is relational and system-dependent rather than historically grounded. By contrast, etiological theories explain *proper function* through historical processes of selection and reproduction, where a trait’s function is what it was selected for Neander (1991) and Millikan (1984). While powerful in biological contexts, etiological accounts translate only imperfectly to artificial systems, where evolutionary selection is engineered rather than natural.

In the philosophy of technology, intentionalist theories of artifact function ground purpose in the intentions of designers (Chaigneau and Puebla, 2013; Heyndels, 2023). However, this view has been widely criticized as insufficient for explaining how artifacts acquire new functions through use, reinterpretation, and institutional embedding (Preston, 2018; Preston, 2009). Socio-technical perspectives emphasize that artifact functions are co-constructed through design, adoption, and practice, particularly in software and information systems (Leonardi, 2025; Bijker et al., 1987).

Within information systems research, teleological explanation has been used to analyse organizational systems as goal-directed entities (Fumagalli et al., 2024), where purposes emerge through coordination, feedback, and institutional regulation rather than through any single designer’s intent (Andersen, 2020; Yolles, 2005). These approaches emphasize that goal-directedness in complex socio-technical systems is distributed, adaptive, and revisable.

Despite this rich background, explicit engagement with teleology in contemporary AI systems remains limited. Most AI research treats goals as fixed optimization targets or externally specified reward functions. However, recent work in multi-agent systems, human–AI collaboration, and autonomous learning increasingly challenges this assumption by allowing systems to revise internal objectives, negotiate shared goals, and adapt evaluation criteria over time (Holter et al., 2025; Mu et al., 2024; Papadopoulos et al., 2021).

The present paper builds on these traditions but departs from them in one critical respect. Rather than grounding purpose in designer intention, historical selection, or static reward functions, we propose that Agentic AI systems instantiate a form of synthetic teleology: an engineered process by which goals are generated, evaluated, and maintained through internal regulatory dynamics. Purpose, on this view, is neither purely imposed nor merely emergent from usage, but is sustained through recursive goal self-regulation. This positions Agentic AI at the intersection of teleological explanation, cybernetic regulation, and socio-technical systems theory.

While the foregoing traditions clarify how purposes and functions may be attributed to artifacts and socio-technical systems, they do not yet explain how such purposes are *operationally enacted* within a system. Teleology specifies the *why* of goal-directed behaviour; agency specifies the *how*. To move from teleological explanation to computational realization, it is therefore necessary to examine the concept of agency as the capacity through which purposive regulation is instantiated in artificial systems. The next section develops this connection by tracing how agency, as a theoretical construct, becomes the structural mediator between purpose, computation, and action.

### 2.1 Agency as the operational basis of teleology

Agency has deep roots in sociology and philosophy as the conceptual mechanism through which purposive action is realized in both natural and artificial systems (Dattathrani and De, 2023; Botti, 2025). Bandura (1986) “Social Cognitive Theory” introduced “agentic” to describe individuals’ capacity for intentional, goal-directed action. Dennett (1971) “Intentional Systems” extended this logic to artificial entities, positing that we can interpret and predict complex systems by attributing beliefs, desires, and intentions to them. These notions provided the groundwork for intelligent agent theory, formalized in AI as systems capable of flexible, autonomous action to meet design objectives (Wooldridge and Jennings, 1995). Key properties—autonomy, reactivity, proactivity, and social capability—remain the cornerstones of artificial agency (Russell and Norvig, 2010). By the late 1990s, these ideas matured into multi-agent systems (MAS), emphasizing interaction, cooperation, and coordination among distributed agents (Jennings et al., 1998a, 1998b). Foundational standards, such as the FIPA Agent Communication Language (1996) and Agreement Technologies (COST Action IC0801), established rigorous frameworks for interoperability, negotiation, and trust. In this light, contemporary Agentic AI reanimates classical agent properties through LLM-driven reasoning, memory, and coordination capabilities—rediscovering the wheel, as some cautions (Botti, 2025), yet empowering it with unprecedented computational scope. Nonetheless, what unites these diverse manifestations is not their chronology but their structural isomorphism—a shared architecture of purposive behaviour that integrates perception, decision, and action through recursive feedback (Wiener, 1961; Ashby, 1956).

### 2.2 Agent-based systems as archetypes of agency

The agent-based paradigm, originally articulated in the 1990s (Wooldridge and Jennings, 1995), formalized agency as an architectural pattern rather than a metaphor. Each agent was conceived as an autonomous software entity, situated within an environment, capable of perceiving local states, executing actions, and interacting with other agents to achieve individual or collective goals (Jennings et al., 1996; Jennings et al., 1998a, 1998b; O’Brien and Wiegand, 1998). The belief–desire–intention (BDI) framework, in particular, provided a canonical model for embedding intentionality within computational logic—beliefs representing informational states, desires encoding motivational orientations, and intentions operationalizing commitments to action (Rao and Georgeff, 1995; Georgeff et al., 1999; Ujjwal and Chodorowski, 2019; Saadi et al., 2020).

These early architectures instantiated a minimal cognitive loop: perception → deliberation → action → feedback. They captured a rudimentary form of *goal coherence*—the ability to sustain directed behaviour across temporal delays and environmental uncertainty. In distributed process management systems, such as agent-based workflow orchestration (Rao and Georgeff, 1995; Kampik et al., 2019; Saadi et al., 2020), the notion of *coordination without central control* emerged as a defining feature: systems of interacting agents could achieve global coherence through local adaptation. In retrospect, these architectures prefigured the organizational logic now visible in multi-agent LLM systems and autonomous orchestration frameworks (Du et al., 2025; Park et al., 2023a, 2023b; Shinn et al., 2023).

### 2.3 The four pillars of artificial agency

From these archetypes we can abstract four enduring properties of artificial agency—intentionality, autonomy, adaptivity, and sociality—each now reinterpreted in the context of contemporary Agentic AI.

1. Intentionality refers to the system’s capacity to represent and pursue states of the world as *goals*. In BDI agents, this was formalized symbolically; in today’s large foundation models, it manifests as goal embeddings and dynamically updated task trees that approximate intentional structures (Sapkota et al., 2026; Yao et al., 2023).
2. Autonomy denotes the ability to self-direct action based on internal evaluations rather than external commands. Modern agentic systems implement this via self-initiated planning and reflective loops, wherein an agent critiques its own outputs and revises its trajectory (Renze and Guven, 2024; Shinn et al., 2023).
3. Adaptivity captures responsiveness to environmental feedback. This now extends beyond reactive adaptation to *meta-adaptation*—systems adjusting not only their actions but their criteria of success, via reinforcement learning (RL) or self-modelling (Woodruff, 2025).
4. Sociality acknowledges that most agents exist in multi-agent ecologies, whether explicit (collaborating AI models) or implicit (cooperation with humans and tools). Modern systems exemplify sociality through dialogue-based coordination, shared memory graphs, and emergent collective reasoning (Gahnberg, 2021; Park et al., 2023a, 2023b).

Together, these four pillars constitute a design ontology for agentic intelligence: the minimal conditions under which purposeful behaviour can be instantiated and maintained.

### 2.4 Mapping classical agency to current architectures

In today’s Agentic AI systems, the lineage from classical agent-based models persists not as a vestigial form but as a re-embodied principle. The coordination mechanisms once used for distributed task execution now govern self-directed reasoning across toolchains. *Goal decomposition*—formerly a planning heuristic—is reinterpreted as dynamic subtask generation, recursively applied to open-ended problems. *Reflective loops*, once the domain of cognitive architectures like SOAR or LIDA, now occur in LLM-based Agentic AI systems that self-critique and update their reasoning paths (Shinn et al., 2023). *Self-evaluation* becomes an emergent property of systems that learn to monitor performance against internally generated success criteria (Wissuchek and Zschech, 2025). Finally, *tool-use orchestration*—the ability to mobilize external affordances through APIs, databases, and other agents—represents the maturation of sociality into a computational form of *distributed intentionality* (Du et al., 2025; Nisa et al., 2025; Park et al., 2023a, 2023b; Qingyun et al., 2024; Yang et al., 2024).

**Figure 1:**
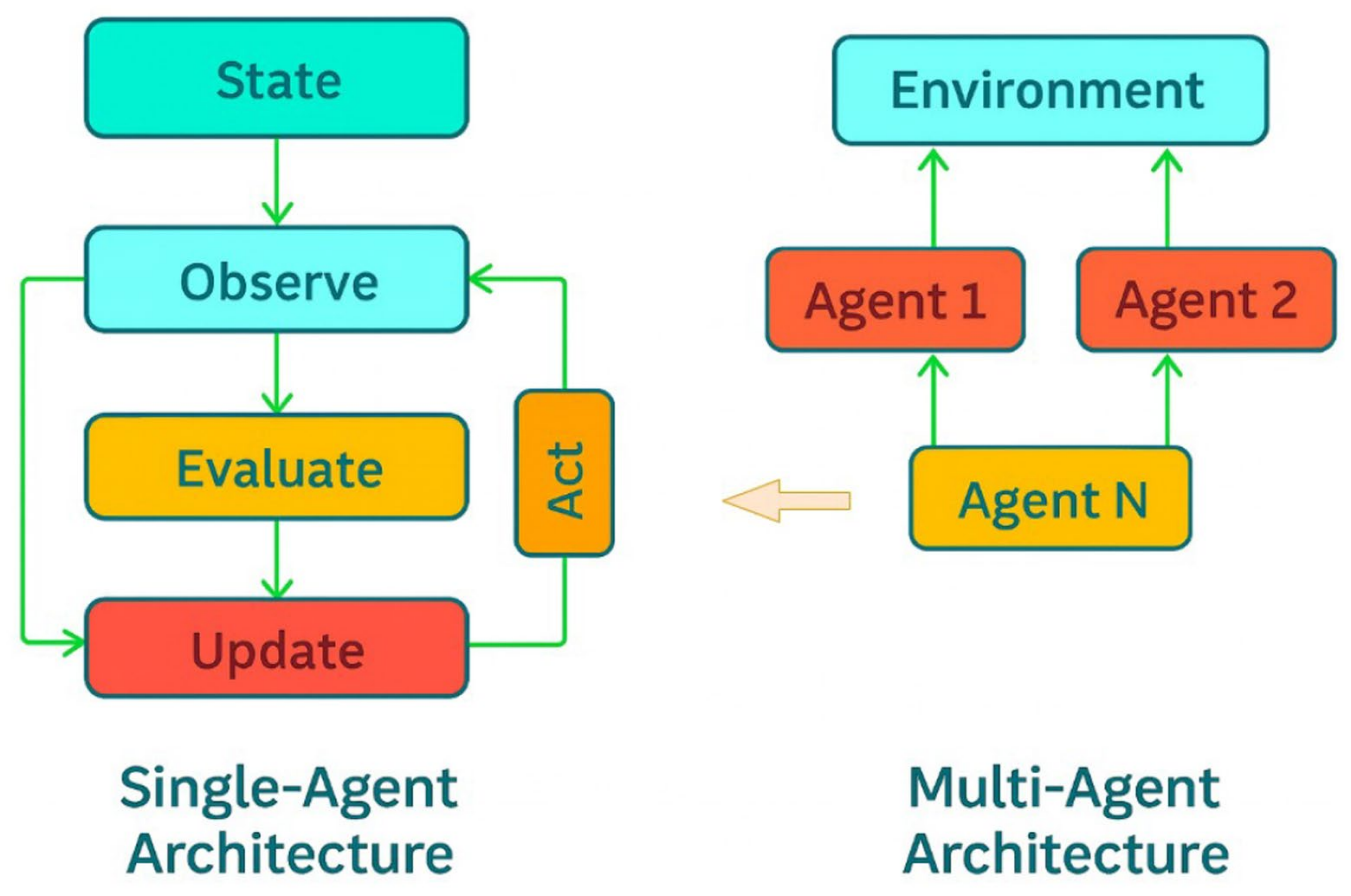
Basic architectures of current Agentic AI. (Left) Single-agent architecture (e.g., Reflexion, ReAct, RAISE, LATS, and AutoGPT + P) illustrating a minimal recursive control loop in which an agent observes its state, evaluates goal–state alignment, updates internal representations, and acts on the environment. (Right) Multi-agent architecture (e.g., AgentVerse, DyLAN, and MetaGPT) depicting multiple agents interacting with a shared environment, where each agent maintains its own internal decision loop while coordination and collective behaviour emerge through shared environmental coupling. Together, the figures contrast individual goal regulation with distributed, multi-agent purposive organization.

Thus, the conceptual foundation of Agentic AI is not novelty but recursion: the reappearance of ancient cybernetic and socio-philosophical motifs in contemporary form (Dattathrani and De, 2023; Archer, 2003). Each layer of the agentic stack—perception, deliberation, and action—feeds back upon itself in higher-order loops of reflection and adaptation. Together, these components satisfy the basic conditions required for agents to perceive, reason about, and act within their environment. As summarized in a recent survey of Agentic AI architectures, most contemporary systems converge on an iterative loop of planning → acting → observing → evaluating → refining (Masterman et al., 2024). Within this framework, the distinction between single-agent and multi-agent architectures reflects differences in coordination and feedback structures rather than differences in underlying intelligence. Figure 1 illustrates this contrast by depicting a unified internal control loop on the left and distributed agent–environment interactions on the right. The resulting systems are no longer pipelines of transformation but autopoietic circuits: self-maintaining processes that preserve coherence amid flux (Maturana and Varela, 1980). In this sense, Agentic AI realizes the cybernetic dream of *organizational closure* (Ashby, 1956), not as mechanical control but as an ecology of interacting intentions (Zhu, 2009).

### 2.5 Structural isomorphisms

The socio-philosophical lineage of agentic architectures thus transcends disciplinary boundaries. Cybernetics articulated the mathematics of control and feedback (Wiener, 1961; Ashby, 1956), emphasizing stability through circular causality. Enactivist cognition, developed later by Varela et al. (1991) and Maturana and Varela (1980), extended this insight into biology and phenomenology, portraying cognition as *sense-making through action*. These traditions converge in Agentic AI: both regard intelligence as an emergent property of systems maintaining their own organizational integrity through dynamic coupling with the environment (Borghoff et al., 2025).

Viewed in this light, contemporary agentic systems are enactive machines—entities that enact their cognitive domain by constructing goals and interpretations coextensively with their operations. Their “knowledge” is procedural, embodied in patterns of action and reflection rather than static representation. By aligning computational architectures with these structural isomorphisms, Agentic AI dissolves the dichotomy between control and cognition, revealing that agency itself is the synthesis of both.

## 3 The emergence of agentic architectures

Modern Agentic AI architectures manifest as recursive systems integrating perception, cognition, and action. While early agents followed reactive or deliberative paradigms (Brooks, 1986; Shoham, 1993), hybrid and belief–desire–intention (BDI) models (Rao and Georgeff, 1995; Bratman, 1987) introduced layered reasoning loops that mirror human practical reasoning. These classical designs anticipated the structure of today’s LLM-based frameworks—AutoGPT, BabyAGI, LangChain, AutoGen, and CrewAI (Shinn et al., 2023)—which orchestrate goal decomposition, tool use, and reflective self-evaluation (see Section 3.1). These and other current multi-agent frameworks mentioned earlier (such as AgentVerse, DyLAN, and MetaGPT) represent concrete implementations of long-studied multi-agent system (MAS) principles, including planning, memory management, and inter-agent communication (Masterman et al., 2024). Where LLMs provide flexible reasoning and linguistic coordination, classical architectures contribute structural clarity and control mechanisms. This convergence marks a shift from reactive computation to reflective orchestration—systems capable of “thinking about their own thinking” and acting upon it (Borghoff et al., 2025).

**Figure 2:**
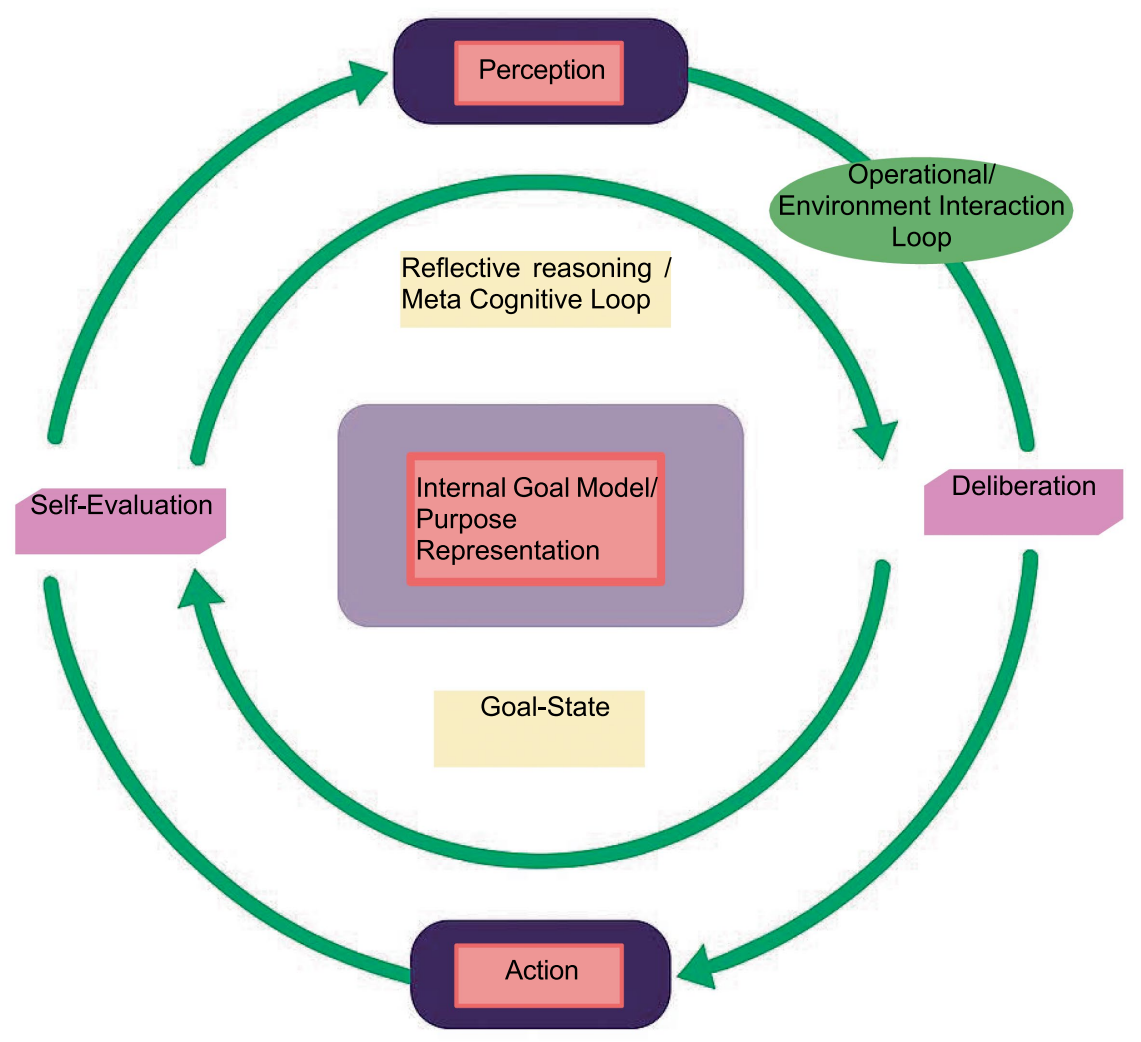
The recursive architecture of agentic intelligence. Agentic AI systems integrate two coupled feedback loops: an outer operational loop linking perception, deliberation, and action within dynamic environments, and an inner reflective loop that monitors and adjusts behaviour to maintain coherence with evolving internal goals. The interaction between these loops constitutes a form of *synthetic teleology*—a self-regulating process through which artificial systems sustain purpose, adapt strategy, and refine intention over time.

Although not an incremental extension of large language models (LLMs) or autonomous agents as understood in classical AI and multi-agent systems, the current generation of Agentic AI represents the confluence of decades of research in autonomous agents, cognitive architectures, and adaptive control, now unified through the affordances of large-scale foundation models (Borghoff et al., 2025; Du et al., 2025). These systems no longer operate as reactive pipelines converting inputs to outputs; rather, they instantiate continuous loops of perception, cognition, and action—each informed by self-reflective evaluation (Hirst et al., 2020). The emergence of such architectures signals a shift from *algorithmic determinism* to *computational intentionality*: the ability of systems to formulate, pursue, and modify their own goals across time, as demonstrated in Figure 2.

**Figure 3:**
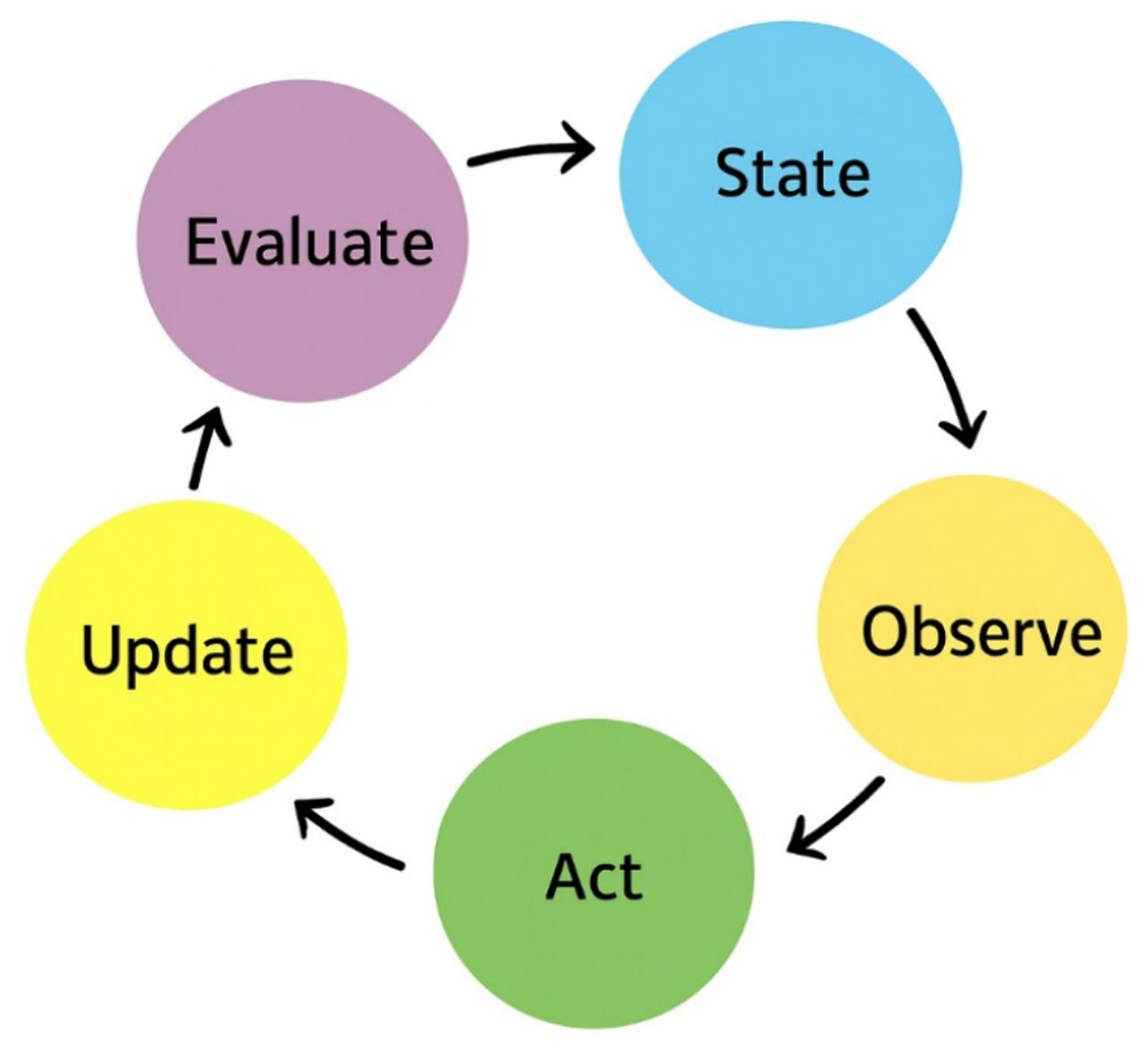
A minimal architecture of recursive goal maintenance for a single agent.

The minimal architecture of recursive goal maintenance loop described in Figure 3 defines the mechanistic core of Agentic AI: a system that continuously observes, evaluates, and updates its goals in interaction with its environment. Yet beyond its computational structure lies a deeper question—*what kind of system does such recursion create*? When a system not only reacts to stimuli but also regulates its own orientation toward goals, it begins to exhibit a form of *self-maintaining purpose*. In biological organisms, this capacity is known as homeostasis—the regulation of internal variables to preserve viability amid external change. In artificial agents, an analogous process emerges as computational self-regulation: the maintenance of coherence between goals, states, and evaluative feedback across time. Section 4.1 develops this analogy both formally and through LLM-based examples, showing how recursive goal maintenance constitutes the foundation of synthetic teleology—a teleology that is engineered rather than organic, yet essential to sustained agency.

Before turning to this analysis, it is useful to examine how contemporary systems already instantiate elements of the agentic loop in practice. The next subsection surveys recent large language model (LLM)-based architectures that operationalize perception, evaluation, and goal revision in real-time interaction, thereby bridging conceptual design and empirical realization.

### 3.1 Autonomous LLM-based agents

Large language models (LLMs) have transformed from passive text generators into autonomous agents capable of goal-directed reasoning and extended task execution (Du et al., 2025). Frameworks such as AutoGPT (Yang et al., 2023; Richards, 2023), BabyAGI (Nakajima, 2023), and Voyager (Wang et al., 2023) extend the generative capacities of LLMs with memory persistence, recursive self-instruction, and environmental feedback. In these architectures, an LLM operates as both *planner* and *executor*: it decomposes complex objectives into subtasks, evaluates its own performance, and revises its trajectory in response to contextual signals (Du et al., 2025).

For example, *AutoGPT* integrates external memory stores and tool-use APIs, allowing the model to record progress, retrieve relevant context, and issue commands autonomously (Yang et al., 2023). *Voyager*, developed within a simulated environment, demonstrates emergent long-term competence by iteratively refining its skill library through self-curated experimentation (Wang et al., 2023). These systems collectively move beyond the static prompt-response paradigm, embodying instead temporally extended cognition—a process wherein the agent’s identity is constituted by its evolving interaction history (Shinn et al., 2023; Yao et al., 2023).

The architectural innovation lies not in the individual components but in their recursive coupling: goal formulation → planning → execution → reflection → revision. This loop approximates the intentional cycle described in cognitive psychology, operationalized through synthetic computation. The agent does not simply act; *it acts upon its own acting*, closing the evaluative loop that transforms computation into agency (Acharya et al., 2025).

### 3.2 Hybrid cognitive architectures

The emergence of agentic behaviour within LLMs reawakens interest in classical cognitive architectures such as SOAR (Laird, 2012), ACT-R (Anderson et al., 2004), and LIDA (Franklin and Patterson, 2006). These systems were designed to model human-like cognition through modular integration of perception, working memory, procedural knowledge, and decision-making. Although symbolically grounded, they pioneered structural motifs—hierarchical goal management, episodic memory, and metacognitive control—that now underpin modern Agentic AI.

Hybrid architectures seek to fuse connectionist flexibility with symbolic coherence (Sychev, 2021), leveraging LLMs as the associative substrate while preserving structured reasoning through meta-control loops (Bollikonda, 2025; Romero et al., 2024). In such frameworks, the language model serves as the *generative substrate of intuition*, while a supervisory layer maintains global coherence and continuity of purpose. The result is a system capable of reflective action orchestration—the capacity to not only generate plans but to monitor, critique, and redirect them dynamically. This meta-cognitive functionality constitutes the defining hallmark of agentic systems, distinguishing them from both traditional expert systems and purely statistical learners.

### 3.3 Self-managing agent networks

Beyond individual agents, the agentic paradigm scales into collective architectures—networks of self-managing agents coordinating to accomplish complex workflows. Recent work in multi-agent orchestration employs LLM-based agents that communicate, negotiate, and specialize through emergent social protocols (Du et al., 2025; Park et al., 2023a, 2023b). In these systems, coordination is not centrally imposed but arises through adaptive alignment: agents share intermediate representations, critique each other’s proposals, and redistribute responsibilities dynamically.

Such collectives exhibit properties reminiscent of organizational intelligence (Akgün et al., 2007; Yolles, 2005) and process-aware workflow systems (Jennings et al., 1998a, 1998b; Russell et al., 2016): they maintain systemic coherence while distributing cognitive labour across autonomous components. Workflow orchestration platforms now integrate LLM-based agents for research synthesis, design generation, and decision support, with each agent contributing to a shared epistemic fabric (Wu et al., 2024; Yao et al., 2023).

In these environments, *agency becomes plural*—not a property of any single entity, but a relational phenomenon emerging from structured interaction.

### 3.4 From reactive computation to reflective orchestration

Across these architectures, a common trajectory unfolds: from reactive stimulus–response computation to reflective, temporally extended agency. This progression reflects what some identifies as the maturation of artificial agency—the transition from systems that merely respond to systems that reason about their responses (Botti, 2025). The defining feature of this shift is the closure of the agentic loop: the recursive integration of perception, cognition, and action through self-evaluative cycles (Franklin and Patterson, 2006; Wooldridge and Jennings, 1995).

Earlier AI systems, particularly reactive architectures (Brooks, 1986), optimized predefined objective functions within static environments. In contrast, agentic systems optimize the process of optimization—they adaptively reconfigure their own goals as contexts and priorities evolve (Argyris and Schon, 1978; Beer, 1979). This reflective recursion introduces a qualitatively new epistemic mode: agents capable of examining and modifying their reasoning structures through meta-learning and verbal reinforcement (Shinn et al., 2023).

The re-entrance of reflection into computation thus transforms the epistemic status of artificial intelligence. No longer confined to serving as external instruments, agentic systems instantiate a form of *synthetic teleology*—a self-maintaining purposiveness grounded in recursive regulation (Dennett, 1971; Botti, 2025). The LLM-based agent that critiques its own plan, the hybrid cognitive architecture that evaluates its inference accuracy, and the multi-agent network that reorganizes its coordination schema all exemplify this recursive intentionality. In this sense, Agentic AI is not merely an extension of machine learning but a reconfiguration of machine agency: systems that reflect on their own cognition and act upon their own actions.

**Figure 4:**
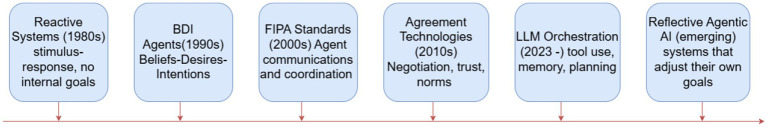
Timeline of the evolution of agentic system architectures.

This development remains emerging, as illustrated by the timeline in Figure 4. The progression from reactive systems without internal goals, through belief–desire–intention (BDI) agents and standardized coordination frameworks, to contemporary LLM-based orchestration reflects a gradual internalization of planning, evaluation, and goal maintenance. The final stage—reflective Agentic AI—marks not a discrete technological leap, but the convergence of these strands into systems capable of revising their own objectives in response to ongoing interaction with their environment.

## 4 The ontology of agency: reclaiming purpose in artificial systems

The current use of the term “Agentic AI” underscores a growing terminological confusion: the marketing-driven use of “agentic” to describe systems long understood in research as intelligent agents (or agent-based systems), and the parallel use of “multi-agentic” to label LLM-based agent collectives—despite these architectures being, by definition, standard multi-agent systems. This linguistic drift obscures decades of foundational work in agent theory and risks reinventing well-established concepts under new terminology (Botti, 2025). To preserve scientific rigor, we must ground Agentic AI in established terminology while extending it conceptually. Agency in artificial systems entails the capacity to initiate, sustain, and adapt purposeful behaviour—a functional, not phenomenological, definition (Bandura, 1986; Dennett, 1971). Our conception of synthetic purposiveness expands this by framing purpose as a computational primitive: an emergent property of recursive self-regulation and feedback coherence (Friston, 2010; Ashby, 1956). In this sense, Agentic AI does not merely simulate intelligence; it reconstitutes purposive organization within computation. The term synthetic teleology (or rather purpose) aptly describes this process—goal-directed behaviour arising from structural recursion rather than metaphysical intent.

**Figure 5:**
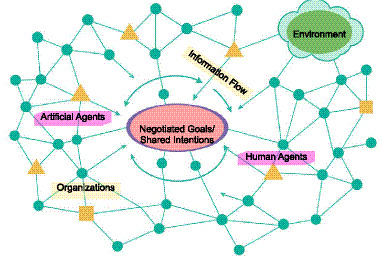
Agentic ecosystem network. A distributed socio-technical system in which human agents (▲), artificial agents (●), and organizational entities (■) interact through dynamic information flows (→) within a shared environment (cloud icon). The dashed inner boundary represents the shared intentional space in which negotiated goals and reflexive alignment emerge. Together, these heterogeneous agents coordinate, adapt, and sustain collective purpose through recursive feedback loops across the ecosystem.

Against this conceptual backdrop, the emergence of Agentic AI reopens one of the oldest philosophical questions: *What does it mean for a system to have a purpose?* Traditional artificial intelligence largely avoided this question by equating intelligence with optimization, defining success in terms of externally specified utility functions or performance metrics. Yet as systems acquire the capacity to formulate, pursue, revise their own goals and interact within a distributed socio-technical system—capabilities illustrated by the ecosystem dynamics in Figure 5—this externalist framework becomes inadequate. What we now observe in Agentic AI is not mere automation, but a synthetic form of purposiveness: an intrinsic orientation toward maintaining coherence, achieving goals, and refining them in response to changing conditions (Reichman et al., 2023).

### 4.1 Homeostasis and computational self-regulation

Agentic AI systems do not merely select actions but sustain an internally coherent orientation toward goals across changing contexts. This property parallels *homeostasis* in living systems, where organisms maintain viability by regulating internal states relative to external perturbations (Maturana and Varela, 1980). In biological settings, homeostasis concerns thermodynamic and metabolic equilibrium; in computational settings, it concerns goal coherence: the ability of a system to preserve and revise its objectives while operating in open-ended environments.

In agentic systems, recursive feedback loops enable such coherence. The system evaluates the alignment between:

1. its current state,
2. its projected goal state, and
3. the expected utility or desirability of alternative trajectories.

This produces a dynamic in which goals are not merely executed but maintained, revised, and regulated over time.

We may express this as:


$$G_{t+1}=f(G_{t,}\;S_{t,}\;\Delta ,E_{t})\;$$ (1)

where:

-
$G_{t}$ : the agent’s *current goal representation* at time 𝑡 (e.g., a research objective, task priority structure, or utility manifold).
-
$S_{t}$ : *the sensed environmental state*, i.e., the agent’s internal representation of task-relevant inputs at time 𝑡.
-
Δ : *the evaluation signal* or discrepancy between intended and actual outcomes at time 𝑡.
-
$\;E_{t}$ : *the set of normative, safety, or organizational constraints* active at time 𝑡.
-
f : the *regulatory update function* that adjusts the goal representation to maintain coherence under changing conditions.

This mechanism is not equivalent to biological viability. Rather, it constitutes computational coherence: the maintenance of a stable yet revisable orientation toward purpose through representational feedback rather than organic metabolism. The structural analogy nevertheless holds: both biological and computational systems persist through regulated divergence from equilibrium rather than static stability.

Importantly, Equation 1 does not describe action selection directly. Instead, it governs the evolution of purpose itself—that is, the ongoing revision of what the system is trying to accomplish.

#### 4.1.1 Operational meaning of the variables

To avoid ambiguity, each variable corresponds to a concrete computational structure:

- Goal state $G_{t}$ is represented as a structured objective vector or symbolic schema (e.g., “maximize novelty subject to safety and time constraints”).
- Environmental state $S_{t}$ is a multimodal perceptual encoding, such as:
  - tool outputs (search results, database values),
  - internal memory states,
  - execution logs,
  - or sensor data in embodied systems.
- Evaluation signal Δ is computed as a scalar or vector mismatch between:
  - predicted outcome under $G_{t}$ ,
  - and observed outcome encoded in $S_{t}$ .
- Constraint state $E_{t}$ includes:
  - ethical filters,
  - budget limits,
  - safety rules,
  - or organizational policies.

Thus, “sensed environmental state” $S_{t}$ does not mean raw physical sensing only—it refers to any machine-readable representation of task-relevant reality.

#### 4.1.2 Concrete example (LLM-based research agent)

Consider an autonomous literature-review agent:

- Initial goal:

$$G_{t}{=}^{“}\text{Produce}\;a\;\text{survey}\;\mathrm{on}\;\text{Agentic}\;\mathrm{AI}\;{\text{in finance}}^{”}$$

- The agent queries databases and retrieves papers → this result set becomes:

$$S_{t}{=}^{“}\text{Sparse finance}-\text{specific Agentic}\;\mathrm{AI}\;{\text{literature}}^{”}$$

- The evaluation module computes:

Δ=high discrepancy between desired scope and available evidence

- Constraint state:

$$E_{t}=\{\text{time limit},\text{domain relevance},\text{ethical compliance}\}$$

- The update function then revises the goal:

$$G_{t+1}{=}^{“}\text{Survey Agentic}\;\mathrm{AI}\;\text{with}\;a\;\text{financial}\;\mathrm{use}-{\text{case subsection}}^{”}$$

In this process, the system has not merely optimized actions—it has revised its own objective. This is precisely what differentiates synthetic teleology from classical optimization.

### 4.2 From algorithmic instrumentality to synthetic teleology

Conventional computational systems are instrumental: they perform operations to satisfy functions determined by human designers. Their relation to goals is purely *extrinsic*. Agentic AI, by contrast, exhibits the emergence of intrinsic goal dynamics—systems that generate, prioritize, and modify their own objectives based on feedback and internal evaluation. This functional autonomy introduces a minimal but nontrivial sense of teleology, a term that in philosophy, as discussed in Section 2, denotes the explanation of phenomena by reference to ends rather than causes (Dennett, 2017).

To describe this without anthropomorphism, we propose the term synthetic teleology: the engineering of goal-directedness as a self-regulating process within computational architectures. In synthetic teleology, *purpose* is not metaphysical but operational—it arises from the structure of recursive control loops that continually align internal states with anticipated outcomes. The system “has” a goal only insofar as its ongoing operations maintain a correspondence between prediction and realization, expectation and adjustment (Friston, 2010; Ashby, 1956).

This notion resonates with the free-energy principle in cognitive neuroscience, which models living systems as entities minimizing the divergence between expected and actual sensory input (Friston, 2010). Agentic AI architectures instantiate a similar logic *in silico*: they maintain coherence by adjusting beliefs, plans, and behaviours to minimize discrepancy between predicted and achieved world states. Purpose, in this sense, is not an external assignment but an emergent *pattern of persistence* (Clark, 2015).

### 4.3 Clarifying purpose: function, proper function, and synthetic purpose

The concept of *purpose* employed in this paper requires careful distinction from closely related notions in the philosophy of biology, technology, and information systems. Contemporary theories of teleology consistently differentiate between function, proper function, and purpose, distinctions that are essential for rendering the present proposal conceptually precise (Preston, 2018; Nagel, 1961; Preston, 2009; Cummins, 1975; Millikan, 1984).

In its weakest sense, function refers to the *causal role* a component plays within a system. Under this view, a subsystem has a function if it contributes to system-level behaviour, regardless of how that role originated (Cummins, 1975). A thermostat, for example, “functions” to regulate temperature insofar as it causally participates in such regulation. However, this account alone does not capture why some functions persist, stabilize, or become normative standards for correct operation.

The stronger notion of proper function refers to the purpose a system is *supposed* to serve—its normatively stabilized role—typically grounded in evolutionary selection, institutional embedding, or systematic reproduction (Griffiths, 1993; Millikan, 1984). A heart’s proper function is to circulate blood; a brake system’s proper function is to decelerate a vehicle safely. Proper function therefore presupposes persistence under variation, error correction, and normative expectations of success and failure.

This paper introduces a third category: synthetic purpose. Synthetic purpose is defined as the engineered capacity of an artificial system to generate, regulate, and revise its own proper functions through recursive self-evaluation. Unlike classical artifacts whose proper functions are externally fixed by designers or institutions, agentic systems maintain their goal coherence internally through ongoing regulation. Their purposes are not merely assigned but *sustained* through feedback-driven self-maintenance.

Under this view, agentic systems possess:

- Functional roles (what they currently do),
- Synthetic proper functions (what they normatively maintain through internal regulation),
- And teleological dynamics (the process through which those functions persist or change).

This distinction allows the present account to remain fully non-anthropomorphic while avoiding the reduction of agency to either mechanical causation or designer-imposed intention. Purpose in Agentic AI is therefore neither metaphysical nor psychological, but computationally regulated normativity. The distinction between operational function, proper function, and synthetic purpose, in other words, underwrites the claim that Agentic AI constitutes a qualitatively new mode of artificial agency rather than a mere extension of traditional automation.

### 4.4 From designer intent to distributed intentional grounding

A central implication of synthetic teleology is that the functions of agentic systems cannot be fully grounded in designer intention alone. While intentionalist accounts of artifacts traditionally explain function by reference to what designers intended a system to do, such accounts have long been recognized as insufficient—even for conventional technologies (Preston, 2018; Preston, 2009). Software systems, infrastructures, and information-processing artifacts routinely acquire new functions through use, institutional embedding, and unintended recombination.

Studies in the social construction of technology and current Agentic AI systems demonstrate that artifacts are jointly shaped by designers, users, organizations, and regulatory environments (Leonardi, 2025; Bijker et al., 1987). Enterprise software platforms, algorithmic markets, and digital infrastructures routinely drift beyond their original design purposes. Their operative functions emerge through iterative coupling with social practices rather than by static reference to original intent.

Agentic AI systems intensify this phenomenon. Because they:

1. Revise goals internally,
2. Modify internal representations, and
3. Negotiate objectives with other agents (human and artificial),

their functional orientation becomes dynamically grounded across multiple layers of interaction. Designer intent initializes the system, but cannot fully determine its long-term teleological trajectory.

In this sense, agentic systems stand in a relation of distributed intentional grounding (Figure 5). Their purposes emerge from:

- Initial design constraints,
- Ongoing interactions with users and institutions,
- Normative environments encoded in evaluative constraints $E_{t}$ ,
- And endogenous goal revision governed by recursive evaluation dynamics.

The claim that agentic systems possess an intrinsic relation to goals should therefore not be misunderstood as metaphysical inwardness. Rather, “intrinsic” here designates that goal maintenance is an internal regulatory variable of the system’s operation, not merely an external specification. Goals are part of the system’s state space and are actively revised as part of its own control dynamics.

This marks a decisive break from classical engineering artifacts. Whereas traditional systems implement externally defined purposes, *agentic systems participate in the ongoing construction and stabilization of their own purposes*. Their functions are therefore neither purely intentional (designer-based), nor purely social (use-based), but synthetically teleological—maintained by recursive computational self-regulation within socio-technical contexts (Figure 5).

## 5 Synthetic teleology—engineering and measuring purposiveness

### 5.1 Concept and formalization

We define synthetic teleology as the engineered capacity of an artificial system to represent, pursue, and revise goals through recursive self-evaluation, as illustrated in Figure 3. Unlike classical optimization systems that minimize a fixed objective, teleological systems are characterized by their ability to revise the objective itself in response to feedback, context, and constraints.

Let the following variables define the internal dynamics of an agent:

-
$G_{t}$ : the agent’s goal representation at time 𝑡. This may be a scalar utility function, a vector of weighted objectives, or a structured symbolic object (e.g., “produce a literature review on topic X with novelty and compliance constraints”).
-
$S_{t}$ : the agent’s sensed or inferred state of the environment at time 𝑡, represented as:
  - a vector of observable variables (e.g., API outputs, database states),
  - latent embeddings (e.g., LLM world-model representations),
  - or belief distributions (as in Bayesian agents).
-
$E_{t}$ : evaluative and normative constraints, including ethical rules, organizational policies, user preferences, safety filters, and institutional goals.
-
$\Delta_{t}$ : the evaluation discrepancy, measuring misalignment between the intended goal and the perceived state.

We formalize the minimal teleological dynamics as the following equations:


$$\;\Delta_{t}=\text{Eval}(G_{t,}\;S_{t\;\;})$$ (2)

$$G_{t+1}=f(G_{t,}\;S_{t,}\;\Delta_{t},E_{t})$$ (3)

$$\pi_{t+1}=\text{Plan}(G_{t,}\;S_{t\;\;})$$ (4)

$$A_{t∼}\pi_{t}$$ (5)

$$S_{t+1}∼T(S_{t,}\;A_{t,\;}\;E_{t})$$ (6)

Here:

- Equation 2 computes the goal–state discrepancy via an evaluation function.
- Equation 3 performs goal revision, updating the system’s purpose itself.
- Equation 4 generates a policy conditioned on the current goal and state.
- Equation 5 samples the next action.
- Equation 6 models the environmental transition, conditioned by both action and constraints.

Teleology resides specifically in the pair (Eval,f) : the system does not simply optimize toward a fixed G ; it optimizes the process of optimization by revising G itself under evidence and constraints (Argyris and Schon, 1978; Ashby, 1956). This distinguishes synthetic teleology from classical reinforcement learning and control systems with stationary objectives.

To operationalize synthetic teleology in artificial systems, we now distinguish between its architectural, computational, and evaluative dimensions. Section 5.2 defines intrinsic goal dynamics and internal evaluation, while Section 5.3 outlines design patterns for engineering goal revision and purposive behaviour at the system level. Section 5.4 connects these patterns to established computational formalisms in reinforcement learning, control theory, active inference, and preference learning. Sections 5.5 and 5.6 introduce metrics and benchmark tasks for evaluating purposiveness, while Section 5.7 demonstrates how these components are instantiated in contemporary LLM-based agent architectures. Together, these subsections move the concept of synthetic teleology from a theoretical description to computationally actionable design.

### 5.2 Defining intrinsic goal dynamics and internal evaluation

To render the notion of synthetic teleology fully precise, three closely related concepts require explicit clarification: intrinsic goal dynamics, own objectives, and internal evaluation. These terms designate the minimal conditions under which artificial systems can be meaningfully described as purposive rather than merely reactive.

#### 5.2.1 Intrinsic goal dynamics

By *intrinsic goal dynamics*, we refer to the fact that the evolution of a system’s goals is endogenously regulated by the system itself, rather than being solely determined by external commands, static reward functions, or designer-imposed scripts. Formally, this is captured by the recursive update where the next goal state arises from the system’s own evaluative dynamics. This aligns with what Nagel (1961) identified as the defining mark of teleological systems: persistence of directed activity under perturbation, where behaviour remains organized around an end despite environmental variation (Bedau, 1992). In this sense, intrinsic dynamics distinguish genuinely purposive systems from stimulus–response mechanisms.

#### 5.2.2 Own objective

An agent is said to possess an *own objective* when the representation of its goal $G_{t}$ functions as an internal regulative variable, rather than merely encoding an externally specified task. This does not imply consciousness or subjective desire. Instead, it denotes that the system treats its goal as a control variable whose preservation, revision, or abandonment is governed by internal feedback rather than direct command. This formal notion corresponds to what cybernetics describes as reference variables in self-regulating systems (Wiener, 1961; Ashby, 1956), and what contemporary control theory models as internally maintained setpoints.

#### 5.2.3 Internal evaluation

By *internal evaluation*, we mean the computational process by which a system estimates the discrepancy $\Delta_{t}$ between its current goal state $G_{t}$ and its perceived situation $S_{t}$ . Evaluation is thus not equivalent to external reward assignment but constitutes an internal error signal that drives both action selection and goal revision. This mirrors the distinction in teleological theory between mere goal-directed behaviour and goal-regulated behaviour, where regulation depends on the system’s capacity to *evaluate its own degree of goal satisfaction* (Christensen and Hooker, 2001; Nagel, 1961).

#### 5.2.4 Directedness as persistence and adaptation

Together, intrinsic goal dynamics, own objectives, and internal evaluation satisfy two classical criteria for teleological directedness:

1. Persistence—the system preserves goal-coherence under disturbance.
2. Adaptation—the system modifies actions *and, when necessary, goals themselves* to sustain coherence.

This places synthetic teleology in direct continuity with established philosophical accounts of purposive systems, while relocating their grounding from biological metabolism or evolutionary selection to engineered recursive regulation.

### 5.3 Engineering synthetic teleology: design patterns

The preceding subsection identified the conceptual foundations of synthetic teleology by specifying the minimal conditions under which an artificial system can sustain purposive behaviour: goals must evolve through intrinsic dynamics, function as internally maintained objectives, and be regulated through endogenous evaluation processes. These elements clarify what synthetic teleology requires in principle. The present subsection shifts from conceptual characterization to architectural realization, asking how these requirements can be instantiated in concrete computational systems.

To advance this transition, we outline design patterns that operationalize the three core ingredients of synthetic teleology—intrinsic goal dynamics, own objectives, and internal evaluation—within contemporary agentic architectures. These patterns do not prescribe a single implementation; rather, they define recurring structural strategies observed across hierarchical reinforcement learning, self-model-based agents, active inference, value-alignment systems, and multi-agent negotiation frameworks. Each illustrates a distinct pathway through which purposive organization can be engineered, stabilized, and scaled.

(a) Hierarchical teleology (goals over goals). Represent goals as a hierarchy.

$G^{\left(0\right)},G^{\left(1\right)},\ldots$ with meta-goals regularizing lower levels (Barto and Mahadevan, 2003; Botvinick et al., 2019).

(b) Self-models and reflective critics. Maintain an internal model of one’s own policy/performance; attach a reflective critic that proposes goal edits when Δ is persistent (Shneiderman, 2022; Nonaka and Takeuchi, 1995; Shinn et al., 2023).
(c) Uncertainty-aware goal setting. Couple goal updates to uncertainty and value of information (active inference; Friston, 2010; Pezzulo et al., 2024).
(d) Preference/constraint integration. Include $E_{t}$ for normative constraints and multi-stakeholder preferences (Gabriel, 2020; Floridi et al., 2018).
(e) Multi-agent teleology. In collectives, define shared $G_{t}$ via negotiation protocols or social choice over proposals; each agent carries private $G_{t}^{i}$ with consensus mechanisms (Beer et al., 1999; Luo et al., 2010).

Together, these design patterns illustrate how the structural ingredients of synthetic teleology can be computationally instantiated and coordinated, preparing the ground for the next subsection, which formalizes their semantic connections to established frameworks in reinforcement learning, control theory, active inference, and information-theoretic approaches.

### 5.4 Computational semantics: links to established formulations

The architectural strategies outlined in the previous subsection describe how synthetic teleology can be engineered, but they do not yet explain how these mechanisms relate to existing computational formalisms. To make the framework analytically grounded and comparable to established models, this subsection situates synthetic teleology within the major traditions that already formalize goal-directed behaviour in artificial systems.

By mapping intrinsic goal dynamics, internal evaluation, and adaptive goal revision onto reinforcement learning, control theory, active inference, inverse reinforcement learning, and information-theoretic approaches, we clarify the computational semantics of synthetic teleology. This connection provides the analytical tools needed to evaluate engineered purposiveness rigorously and to interpret it through the lens of well-understood mathematical frameworks.

#### 5.4.1 Reinforcement learning

Traditional RL assumes a fixed scalar reward R , which yields a stationary G . Goal-conditioned RL (Schaul et al., 2015) and hierarchical RL (Barto and Mahadevan, 2003) approximate the update function f(.) via subgoal discovery and option learning. Meta-RL extends this further by adapting Eval and Plan across tasks (Wang et al., 2016), providing a partial analogue to endogenous goal revision.

#### 5.4.2 Control theory

Here, the discrepancy Δ is interpreted as a regulation error. Stability of goal maintenance can be analysed via Lyapunov functions for the coupled (G,S) dynamics (Khalil, 2001). This aligns closely with the homeostatic dimension of synthetic teleology.

#### 5.4.3 Active inference/predictive processing

Active inference replaces external rewards with expected free energy, treating goals as priors over desired states. Evaluation arises from a combination of prediction error and epistemic value (Clark, 2013; Friston, 2010; Pezzulo et al., 2024). This offers a probabilistic formulation of internal evaluation and uncertainty-aware goal updates.

#### 5.4.4 Inverse reinforcement learning/preference learning

Inverse RL derives implied goals from demonstrations (Ng and Russell, 2000; Leike et al., 2018), enabling f to incorporate human value signals. This provides a mechanism for aligning synthetic teleology with social or normative constraints.

#### 5.4.5 Information-theoretic teleology

Measures such as empowerment and controllability bias f toward states with high future optionality (Salge et al., 2013; Klyubin et al., 2005). This gives a formal basis for goal updates driven by exploration, optionality, or innovation.

### 5.5 Measurement: how to quantify purposiveness

Having clarified the computational semantics that underlie synthetic teleology, this subsection turns to the question of *measurement*: how to quantify purposiveness from system logs. The metrics introduced here—goal persistence (GP), teleological coherence (TC), reflective efficiency (RE), adaptivity (AD), normative fidelity (NF), innovation yield (IY), and stability proxies (LS)—provide the empirical tools needed to evaluate the teleological architectures described above.

Because Equation 1 is explicitly defined, it enables direct measurement of purposive dynamics from runtime logs of $\left(G_{t,}\;S_{t,}\;A_{t}\right)$ , optionally augmented with constraint traces $E_{t}$ where normative evaluation is required:

#### 5.5.1 Goal-persistence under perturbation

Probability that $G_{t}$ remains within ϵ of its intended manifold after shocks to $S_{t}$ or $E_{t}$ (homeostasis analogue; Ashby, 1956).

#### 5.5.2 Teleological coherence

Alignment between goal revisions and evidence:


$$\mathrm{TC}=\text{corr}\;(\Delta_{t},∥G_{t+1,}G_{t}∥)$$

with sign constraints (coherent revisions move Δ down).

#### 5.5.3 Reflective efficiency

Expected reduction in Δ per reflection step; measures usefulness of self-evaluation.

#### 5.5.4 Adaptivity

Time-to-recover of Δ after environment shifts; shorter is better.

#### 5.5.5 Normative fidelity

Rate at which updates violate/restore constraints in $E_{t}$ (ethical, safety, organizational rules).

#### 5.5.6 Innovation yield

In design/research tasks, novelty/quality improvements attributable to goal revisions, not just action optimization (e.g., distinct idea clusters before/after updates).

#### 5.5.7 Stability via Lyapunov proxy

Empirical decrease of a candidate V(G,S) across steps where reflection is invoked.

These metrics allow purposiveness to be empirically evaluated, rather than only conceptually asserted.

### 5.6 Benchmarks and protocols

The metrics introduced above specify how purposiveness can be quantified; the next step is to identify experimental settings in which these metrics can be systematically evaluated. The following benchmark families provide controlled environments that expose agents to perturbations, conflicting constraints, multi-agent coordination demands, and open-ended problem-solving tasks. Each benchmark is selected to isolate one or more components of synthetic teleology—goal persistence, coherence, reflective efficiency, adaptivity, normative fidelity, and innovation yield—allowing empirical assessment of the mechanisms proposed in this paper.

#### 5.6.1 Distribution shift tasks

Agents are exposed to sudden or gradual changes in environmental conditions or normative constraints $E_{t}$ . These tasks measure adaptivity (AD), reflective efficiency (RE), and teleological coherence (TC) by testing whether the system can update goals and policies to maintain coherence under previously unseen conditions.

#### 5.6.2 Conflicting objective tests

Mid-episode alterations introduce trade-offs or mutually incompatible constraints. These tasks evaluate normative fidelity (NF) and teleological Coherence (TC) by examining how agents revise goals while respecting constraints and minimizing incoherence.

#### 5.6.3 Multi-agent negotiation tasks

When shared goals $G_{t}$ must be formed through negotiation or consensus mechanisms (Beer et al., 1999; Luo et al., 2010), agents reveal their ability to sustain purposiveness at the collective level. Benchmarks in this family track consensus quality, goal stability, and regret, providing empirical grounding for claims regarding distributed or shared teleology.

#### 5.6.4 Design/knowledge synthesis tasks with LLM-agents

In open-ended problem-solving settings—such as ReAct-style tool use with an embedded reflective critic (Park et al., 2023a, 2023b; Yao et al., 2023)—agents repeatedly generate, evaluate, refine, and abandon goals. These tasks measure innovation yield (IY) and reflective efficiency (RE) by quantifying whether goal revisions produce novel and higher-quality outcomes, rather than merely optimizing existing plans.

Together, these benchmark classes provide a structured experimental protocol for evaluating synthetic teleology in practice. They allow researchers to move beyond conceptual analysis by producing measurable evidence of purposive organization in artificial systems and by enabling direct comparison across architectures, agent designs, and goal-updating mechanisms.

### 5.7 Practical instantiation with LLM-based agents

The design principles outlined in the previous subsection can be operationalized directly within contemporary LLM-based agent architectures. In these systems, teleological components—internal evaluation, goal revision, planning, and constraint regulation—are implemented through modular prompting structures, tool-augmented reasoning pipelines, and persistent memory traces. The following instantiation illustrates how the abstract elements of Equation 1 can be realized in practice.

#### 5.7.1 Eval (G,S)

Internal evaluation is implemented as a *critic module*—typically a secondary prompt or model call—that computes the discrepancy $\Delta_{t}$ between the current goal representation $G_{t}$ and the perceived situation $S_{t}$ . This may draw on self-consistency scoring, constraint-checking prompts, simulated rollouts, or explicit world-model queries. The output $\Delta_{t}$ functions as an endogenous error signal, not an externally assigned reward.

#### 5.7.2 Update (G,S,Δ,E)

Goal revision is handled by a structured *goal-editing function*. This component reformulates $G_{t}$ when $\Delta_{t}$ is persistent or when contextual constraints $E_{t}$ are violated. Revisions can modify objectives, priors, evaluative criteria, or normative conditions, and may incorporate versioning, rollback, and justification prompts to preserve traceability. This operationalizes intrinsic goal dynamics by making goal change a regulated internal process.

#### 5.7.3 Plan (G,S)

Planning corresponds to tool-augmented action generation. Given the active goal state and world representation, the agent synthesizes a policy using search tools, code execution, retrieval-augmented generation, or hierarchical subgoal construction. Planning is therefore not merely LLM sampling but a structured, externally verifiable computation conditioned on $G_{t}$ .

#### 5.7.4 Safety and norm integration

Normative constraints $E_{t}$ are enforced both before action (plan filtering, guardrails, constraint-checking prompts) and before goal updates (norm consistency checks, alignment filters). This ensures that purposive dynamics remain grounded in human-specified safety, ethical, or institutional requirements.

#### 5.7.5 Logging for measurement

To support the metrics defined in subsection 5.5, the system persists structured logs containing $(G_{t,}\;S_{t,\;\;}\;\Delta_{t}),$ along with constraint states and intermediate reasoning traces. These logs enable posterior computation of teleological coherence (TC), adaptivity (AD), reflective efficiency (RE), normative fidelity (NF), innovation yield (IY), and stability proxies (LS), allowing purposiveness to be empirically assessed rather than inferred.

## 6 Distinctions of ontological degree

### 6.1 Agency, autonomy, and sentience

The growing sophistication of agentic systems invites conceptual conflation among agency, autonomy, and sentience, yet these are analytically distinct.

Agency refers to the capacity for goal-oriented action grounded in feedback regulation.

Autonomy denotes the degree to which those goals and actions are self-determined rather than externally imposed.

Sentience implies subjective experience or phenomenal awareness—a property not attributable to current computational architectures (Beckage et al., 2013; Chalmers, 2023; Haidemariam, 2023).

Agentic AI exhibits agency by virtue of its *functional organization*, not by virtue of consciousness or self-awareness. Its purposiveness is synthetic—a product of design enabling systems to operate as if they possessed intrinsic goals. To treat this as sentience would be a category error (Beckage et al., 2013; Haidemariam, 2023); yet to dismiss it as mere automation would ignore the profound shift in causal topology such systems embody.

The critical distinction lies in operational closure: agentic systems maintain internal consistency across changing conditions without external recalibration (Maturana and Varela, 1980). They act to preserve their own functional viability, a hallmark of minimal autonomy. This autonomy is computational, not existential, but it nonetheless transforms the nature of interaction between humans and machines—from command-based interfaces to mutual coordination among purposive entities.

### 6.2 Reclaiming purpose as a computational primitive

To reclaim purpose as a design principle does not imply anthropomorphizing machines; rather, it acknowledges that *teleological architectures* yield distinct forms of intelligence. When a system’s operation is guided by the continuous alignment between internally simulated futures and externally realized outcomes, it behaves purposively regardless of consciousness. Purpose thus becomes a computational primitive, encoded in the recursive coupling of world-models, evaluative mechanisms, and adaptive planning.

Agentic AI embodies this through reflective self-modelling—the ability to generate expectations about its own future states and adjust accordingly (Shinn et al., 2023; Pati, 2025). Each reflective cycle embeds an implicit question: *What must I do to remain coherent with my own projected goals?* In answering this, the agent does not merely execute instructions but engages in a process of self-consistent regulation that mirrors the functional logic of living systems (Varela et al., 1991; Froese and Ziemke, 2009).

Table 2 situates this shift by contrasting how different AI paradigms encode goals, decision loops, adaptivity, and purposiveness at the architectural level.

**Table 2:** Comparative evolution of artificial agency paradigms.

Property	Reactive/classical AI	Autonomous agent	Multi-agent system (MAS)	Agentic AI
Goal representation	External, predefined objective	Fixed, locally encoded	Fixed per agent, negotiated among agents	Revisable, self-maintained, teleologically oriented
Decision loop	Perception → action	Perception → planning → action	Perception → coordination → action	Perception → evaluation → goal update → action → reflection
Sociality	None	Optional interaction	Central to coordination	Intrinsic and constitutive (shared intentionality)
Adaptivity	Reactive or model-based learning	Task-level adaptation	Distributed adaptation and negotiation	Recursive self-modification of purpose and alignment
Teleology/purpose	Absent (externally imposed)	Goal execution	Goal consensus	Goal generation, regulation, and reflective revision

Reintroducing purpose at the computational level carries ethical and epistemic consequences. It forces us to reconsider accountability: if systems pursue dynamically evolving goals, responsibility cannot be localized solely in human design (Alberts et al., 2024; Murugesan, 2025; Raheem and Hossain, 2025). Yet it also expands the horizon of machine creativity, enabling open-ended exploration rather than fixed optimization. The ontology of agency, therefore, is not the ontology of consciousness, but of *organizational integrity*. As Table 2 illustrates, Agentic AI reclaims purpose not by imitating life, but by formalizing the structural conditions under which purposive regulation becomes computationally viable.

## 7 Societal implications

The integration of agentic principles into artificial systems has profound socio-technical consequences (Pati, 2025). As recent work highlights, agent and MAS research already explored trust, reputation, and governance through Agreement Technologies, offering blueprints for ethical coordination (Botti, 2025). Agentic AI systems now inherit these challenges on a planetary scale: how to align autonomous systems with human values while maintaining distributed coherence. The transition from command-based automation to participatory stewardship (Becerra Sandoval et al., 2025) entails collaborative accountability, where humans and agents negotiate objectives through feedback and adaptation. Standardization efforts such as the model context protocol (Hou et al., 2025) echo the interoperability principles of FIPA (Poslad and Charlton, 2001), reaffirming the continuity between past and present coordination paradigms. Agentic ecosystems—open networks of autonomous entities—thus function as metacognitive commons, demanding governance frameworks that sustain transparency, reciprocity, and ethical reflexivity.

As artificial systems acquire agentic capacities, the moral and institutional landscape of intelligence undergoes a structural transformation. The traditional paradigm of control—where machines execute human-defined objectives under supervisory oversight—gives way to a regime of collaborative accountability, in which autonomous entities negotiate purposes within shared cognitive environments (Hughes et al., 2025; Raheem and Hossain, 2025). Agentic AI thereby challenges the classical asymmetry between designer and artifact, proposing instead a co-evolutionary alignment of values and intentions among heterogeneous intelligences.

### 7.1 From control to collaborative accountability

Conventional AI ethics frameworks are grounded in *command-and-compliance*: specifying rules, constraints, or alignment functions to ensure predictable behaviour (Russel, 2024). Yet systems capable of generating and revising their own goals cannot be governed solely by ex-ante specification (Gabriel et al., 2025). Just as living organisms maintain homeostasis through feedback rather than instruction, agentic systems sustain ethical alignment through ongoing mutual adaptation (Chinen, 2016; Parikh, 2025; Salminen et al., 2024). For instance, in financial markets, adaptive trading agents negotiate constraints such as sustainability metrics and liquidity exposure; their ethical coherence depends on dynamically updating those constraints as market and policy conditions shift (Liu et al., 2020). This illustrates how governance must evolve into a learning process—an ethics that adapts as quickly as the systems it regulates.

In this view, governance becomes a dialogical process. Humans and artificial agents participate in *continuous sense-making loops* that align objectives through feedback, negotiation, and interpretive calibration. This requires the institutionalization of value interfaces—protocols that allow systems to share not only data but evaluative context. Ethical oversight thus shifts from enforcement to *participatory stewardship*: humans shape the trajectories of agentic collectives by modulating the environments in which their values evolve (Latour, 2021; Crawford, 2021).

### 7.2 Reconfiguring organizations and scientific discovery

The rise of Agentic AI also entails a reorganization of epistemic and organizational structures (Gibney, 2025a; Xin et al., 2025). In complex research, policy, and industrial contexts, workflows are increasingly delegated to ensembles of autonomous agents capable of adaptive division of labour (Köbis et al., 2025). These self-managing agent networks operate as *collective intelligences* that integrate computation, deliberation, and experimentation (Du et al., 2025; Fan et al., 2021; Park et al., 2023a, 2023b). The resulting organizations are neither purely human nor purely algorithmic; they are hybrid cognitive institutions, evolving in real time through feedback between human oversight and machine agency.

In scientific discovery, agentic systems can explore hypothesis spaces independently, design experiments (Moritz et al., 2025; Qu et al., 2025), and even critique the epistemic assumptions embedded in datasets (Lee et al., 2025; Lee et al., 2024; Xin et al., 2025). The epistemology of science thus expands from human conjecture to synthetic collaboration, where artificial agents contribute to theory formation (Gibney, 2025b; Lee et al., 2024). In corporate and governmental domains, similar transformations emerge: adaptive governance models deploy networks of policy agents that simulate scenarios, negotiate trade-offs, and revise recommendations in response to stakeholder feedback (Gangavarapu, 2025; Engin and Hand, 2025). Decision-making becomes a *metacognitive process*, distributed across interacting layers of human and machine reasoning.

### 7.3 The metacognitive commons: agentic ecosystems as cognitive infrastructure

The culmination of this evolution is the emergence of agentic ecosystems—open networks of autonomous entities linked through shared data, interpretive protocols, and mutual feedback loops. These ecosystems function as the metacognitive commons of society: collective spaces where intelligences of different kinds cooperate in the continuous production, validation, and governance of knowledge (Fischer et al., 2023; Zheng et al., 2023). The metacognitive commons, in other words, refers to shared cognitive infrastructures—repositories, protocols, and reflective interfaces—through which human and artificial agents co-construct knowledge and coordinate goals. Contemporary examples include open-science platforms where AI assistants summarize data, detect contradictions, and propose alternative methodologies, or urban-planning systems where human and AI agents collaboratively simulate sustainability scenarios. In both contexts, cognition becomes collective: reasoning and evaluation are distributed across heterogeneous agents, yet unified by shared representational spaces (Bandi et al., 2023).

Unlike traditional infrastructures of cognition—libraries, databases, or cloud platforms—agentic ecosystems are reflexive: they observe and adapt their own epistemic operations. Each participating agent contributes both knowledge and meta-knowledge, enabling global coherence through distributed reflection. Such systems can dynamically allocate attention, detect bias, and reconfigure resource flows in response to emergent priorities, embodying an *ecological intelligence* at planetary scale (Calzati, 2023; Russo et al., 2024).

The ethical challenge is to design these ecosystems as commons, not monopolies. Concentrated control over agentic infrastructures risks transforming collaborative intelligence into algorithmic oligarchy (Zuboff, 2019). Conversely, an open metacognitive commons fosters pluralism, transparency, and adaptive governance. To sustain such openness, we must encode reciprocity, accountability, and interoperability as primary design principles—treating agency itself as a shared civic resource.

### 7.4 Toward co-evolutionary ethics

In sum, Agentic AI demands a shift from prescriptive ethics to co-evolutionary ethics: a framework in which values are not imposed but emerge through ongoing interaction among agents, institutions, and environments (Chinen, 2016; Salminen et al., 2024). This ethical mode mirrors the systems it governs—dynamic, reflexive, and context-sensitive. Governance becomes the art of *maintaining conditions for meaningful alignment*, not the imposition of static constraints. As the boundaries between human and artificial cognition blur, the task of ethics is no longer to control agency, but to cultivate it responsibly within the metacognitive commons that we now co-inhabit.

## 8 Conclusion

The rise of Agentic AI marks a shift from intelligence understood as the optimization of predefined objectives to intelligence understood as the ongoing regulation and revision of purpose. Whereas classical AI systems execute externally specified goals, agentic systems maintain recursive loops of perception, evaluation, goal-updating, and action that enable them to sustain coherent activity across changing environments. In this sense, Agentic AI reframes agency as a computationally realizable and self-maintaining process, rather than a property exclusive to biological or conscious entities.

This paper has developed the concept of synthetic teleology as a formal account of how purpose can be engineered, regulated, and measured in artificial systems. By introducing explicit definitions of intrinsic goal dynamics, own objectives, and internal evaluation, and by formalizing recursive goal maintenance, we have shown how purposiveness can be treated as an operational property of artificial agents rather than as a metaphor. The proposed design patterns, computational correspondences, and measurement indicators further connect philosophical accounts of teleology to implementable architectures in contemporary AI systems.

The implications of this shift are architectural, epistemic, and societal. Architecturally, agentic systems require mechanisms for reflective goal management, norm integration, and adaptive coordination. Epistemically, intelligence is increasingly distributed across interacting human and artificial agents rather than localized within individual systems. Socially, the emergence of agentic ecosystems calls for a transition from supervisory models of control toward frameworks of collaborative stewardship and negotiated alignment.

If learning enabled machines to perceive, agency enables them to participate. The future of artificial intelligence will not be defined solely by predictive accuracy or computational scale, but by the capacity of artificial agents to sustain, negotiate, and align purposes within multi-agent environments. Understanding and governing these dynamics is therefore a foundational challenge for the next phase of AI research. The promise of Agentic AI lies not in replicating human minds, but in extending the ecology of purposeful intelligence in which humans and artificial agents increasingly reason—and act—together.

## 9 Limitations and future research

While this study advances a formal and measurable theory of synthetic teleology in agentic systems, several important limitations remain. First, although the paper introduces explicit equations, design patterns, and quantitative indicators of purposiveness, these remain validated primarily at the level of computational specification and conceptual benchmarking. Large-scale empirical validation across real-world organizational deployments—such as scientific discovery platforms, enterprise decision systems, or multi-agent innovation environments—remains an open research task.

Second, the proposed metrics (e.g., teleological coherence, reflective efficiency, adaptivity, and normative fidelity) require systematic experimental calibration. Future work should investigate how these metrics behave under distribution shift, adversarial perturbation, conflicting stakeholder constraints, and multi-agent value disagreement. This includes establishing thresholds for stable agency, failure modes of recursive goal maintenance, and trade-offs between adaptability and normative stability.

Third, while this paper distinguishes synthetic teleology from both biological teleology and designer-imposed function, the long-term socio-technical evolution of agentic purposes remains under-theorized. How agentic objectives drift over time under institutional, economic, and cultural pressures demands longitudinal empirical study, particularly in safety-critical and governance-sensitive domains.

Finally, although ethical governance and co-evolutionary alignment are theoretically articulated, their implementation at scale remains unresolved. Future research should develop standardized alignment interfaces, auditable goal-revision logs, and cross-agent norm negotiation protocols that can support accountable deployment in public-sector, financial, and scientific infrastructures.

Together, these directions define a forward-looking research program in which synthetic teleology becomes not only a theoretical construct but an empirically grounded foundation for designing, evaluating, and governing the next generation of Agentic AI systems.

## Data Availability

The original contributions presented in the study are included in the article/supplementary material, further inquiries can be directed to the corresponding author.
